# Ensemble Learning Prediction of Drug-Target Interactions Using GIST Descriptor Extracted from PSSM-Based Evolutionary Information

**DOI:** 10.1155/2020/4516250

**Published:** 2020-08-21

**Authors:** Xinke Zhan, Zhuhong You, Changqing Yu, Liping Li, Jie Pan

**Affiliations:** School of Information Engineering, Xijing University, Xi'an 710123, China

## Abstract

Identifying the drug-target interactions (DTIs) plays an essential role in new drug development. However, there still has the limited knowledge of DTIs and a significant number of unknown DTI pairs. Moreover, the traditional experimental methods have inevitable disadvantages such as high cost and time-consuming. Therefore, developing computational methods for predicting DTIs is attracting more and more attention. In this study, we report a novel computational approach for predicting DTI using GIST feature, position-specific scoring matrix (PSSM), and rotation forest (RF). Specifically, each target protein is first converted into a PSSM for retaining evolutionary information. Then, the GIST feature is extracted from PSSM and substructure fingerprint information is adopted to extract the feature of the drug. Finally, combining each protein and drug features to form a new drug-target pair, which is employed as input feature for RF classifier. In the experiment, the proposed method achieves high average accuracies of 89.25%, 85.93%, 82.36%, and 73.89% on *enzyme, ion channel, G protein-coupled receptors (GPCRs), and nuclear receptor,* respectively. For further evaluating the prediction performance of the proposed method, we compare it with the state-of-the-art support vector machine (SVM) classifier on the same golden standard dataset. These promising results illustrate that the proposed method is more effective and stable than other methods. We expect the proposed method to be a useful tool for predicting large-scale DTIs.

## 1. Introduction

Identification of drug-target interaction (DTI) plays a vital role in researching and developing new drugs. Recently, many researchers have conducted extensive research into the DTI due to its essential role in seeking new protein to the target for drug development and promoting the emergence of new drug candidates [1, 2]. However, the knowledge structure of drug-target is still incomplete, and only a small portion of target proteins of drugs have been proved as interactive. Researchers have carried out a large number of experimental methods to identify drug-target interactions, but these experimental methods have inevitable shortcomings such as time-consuming and high cost. It is known that drug development is a long process, and the whole process of introducing a new drug to market will take at least more than ten years and cost more than billions of dollars. The Food and Drug Administration (FDA) approved only a few of the drug candidates to reach the market [3, 4], due to many new drug candidates fail to achieve expected performance or have harmful side effects in clinical trials. Therefore, it is becoming increasingly urgent to identify drug-target interactions by developing effective new computational methods [5] which can reduce the cost and time of the experimental approach. The reliable computational method could accelerate drug discovery and potentially find some better drug candidates [6].

With the rapid development of genomics and bioinformatics, the accumulation of drug-target data is increasing. In order to store and apply data more efficiently, many related databases such as Therapeutic Target Database (TTD) [7, 8], Kyoto Encyclopedia of Genes and Genomes (KEGG) [9], SuperTarget and Matador [10], and DrugBank [11, 12] have been established. These massive data can provide abundant resources for researchers to study drug-target interactions and develop a novel computational approach.

Up to now, traditional calculation methods are mainly composed of ligand-based methods [13] and structure-based methods [14–16]. As for ligand-based prediction methods, it is mainly used to predict the biological activity of molecules on specific targets. However, the performance of the constructed model does not satisfy the expected requirements if the number of known active molecules of a specific target is insufficient, and the information of protein domain is unused. For the structure-based method, molecular docking is one of the most widely used methods. This method needs to study the interaction between drug molecules and target proteins through the three-dimensional (3-D) structure information of known targets. At the same time, for a given drug or new chemical entity, reverse docking can be used to predict potential targets with which it interacts [17–19]. However, it is known that proteins with the 3-D structure only account for a small part of the whole proteins, which makes the method difficult to meet the requirements of experimental methods. Therefore, it is more effective to develop new protein sequence-based prediction models to predict drug-target interactions.

Until now, a number of computational methods aimed at identifying new drug-target interactions have been proposed. For example, Yamanishi et al. [20] proposed a bipartite graph learning model method, which integrates the chemical space of drugs, the gene space of proteins, and the topological information of drug target interaction network into a unified pharmacological space. Li et al. [21] proposed a novel prediction method using local binary pattern (LBP) and discriminative vector machine (DVM) for predicting DTIs. Liu et al. [22] proposed a logistic matrix factorization algorithm based on neighbour regularization, using a neighbour regularization factor to solve the problem of new drug additional. Nagamine and Sakakibara [23] developed a computational method which combined the amino acid sequence data, the chemical structure of the ligand, and mass spectrometry (MS) as input data and used the support vector machine (SVM) to build a prediction model. Meng et al. [24] reported a novel computational method, namely, PDTPS, which is aimed at predicting drug-target interactions based on protein sequences and drug chemical structures. Yu et al. [25] designed a relatively systematic method, which integrates the chemical, genomic, and pharmacological information of drugs and targets. Huang et al. [26] proposed a new computational approach for predicting DTIs. Specifically, the protein sequence is transformed into a pseudo substitution matrix representation (Pseudo-SMR) descriptor, which retained the biological evolutionary information and predicting DTIs after connecting two vector spaces of drug structure and protein sequence. In order to handle the problem of imbalance data, many computational methods [27] have been proposed which aims to solve the problem for predicting DTIs. For example, Mahmud et al. [28] presented a new computational model, namely, pdti-EssB, which constructed a predictive model with XGBoost; the model used data-balancing techniques to handle the imbalance problem and adopted a novel feature eliminator for accurate prediction. Ezzat et al. [29] proposed a novel method which is focused on addressing two imbalance problems. The first was solving the high imbalance ratio between the minority and majority classes, and the second was aimed at dealing with the within-class imbalance prevalent; the method is effective for predicting drug-target interactions.

In our work, we proposed a novel computational method, which based on drug substructure fingerprints and the information of the target protein sequence to predict drug-target interactions on a large scale. The proposed method combines GIST feature, position-specific scoring matrix (PSSM), and rotation forest (RF). The method mainly contains three steps: converting the target protein sequence into PSSM and adopting molecular substructure fingerprints as the feature of drugs are the first step, and then GIST feature vectors are extracted from PSSM. Finally, the GIST feature vectors would input to the RF classifier and obtain the result of prediction. In order to better evaluate the proposed method, a five-fold cross validation method is adopted on four golden datasets, including *enzyme, ion channels, GPCRs, and nuclear receptors*. Furthermore, we make a comparison between the proposed method and the state-of-the-art support vector machine (SVM) classifier on enzyme dataset, and we also compare the result of the proposed method with previous work on four datasets. The promising results show that our method is efficient and robust to predict drug-target interactions.

## 2. Materials and Methods

### 2.1. Golden Standard Datasets

In this study, four golden standard datasets, including enzymes, ion channels, GPCRs, *and* nuclear receptors were explored by using the proposed method for evaluating the prediction ability of drug-target interactions. All these datasets were freely available from BRENDA [30], DrugBank [11], KEGG BRITE [9], and SuperTarget [10] databases, and these drug-target datasets from high-reliability databases are generally considered the golden standard datasets. The number of drugs known to target *enzyme*, *ion channels*, *GPCRs*, and *nuclear receptors* is 445, 210, 233, and 54, respectively. The number of proteins targeted by the drugs is known to be 664, 204, 95, and 26, respectively. In these total datasets, 5127 drug-target pairs were known to interact with each other. These data are distributed over *enzyme*, *ion channels*, *GPCRs*, and *nuclear receptors*, respectively, and the number of them are 2926, 1476, 635, and 90, respectively. Table.  1 summarizes the statistics of the number of four drug-target datasets.

Generally speaking, we usually consider a drug-target interaction network as a bipartite graph in which nodes describe drug molecules or target proteins and edges represent the relationship between the nodes. It was very sparse of the initial bipartite graph of drug-target interactions for which only a small fraction of the real drug-target interactions edges have been validated by experimental method or other ways. Take the *enzyme* dataset as an example, and there are 295,480 (445 × 664) connections in the corresponding bipartite graph in total. However, only 2926 initial edges which account for only 1.00% of the total connections were known drug-target interactions. These known interaction pairs were treated as positive samples (2926) which were obviously less than the possible number of negative samples (295, 480 − 2926 = 292,554). There exists a bias problem caused by the unbalance samples. In order to deal with this problem, the number of negative samples was selected randomly as much as the positive samples. It would know that the real interaction negative samples we have chosen is quite small or even can be ignored when study a large-scale of DTI. As a result, the negative samples of *enzyme*, *ion channel*s, *GPCRs*, and *nuclear receptors* datasets were 2926, 1476, 635, and 90, respectively.

### 2.2. Molecular Substructure Fingerprint of Drug

Many kinds of research have shown that drug compounds could be represented as different types of descriptor such as constitutional, topological, quantum chemical properties and geometrical. Here, molecular fingerprints are being employed to represent drug compounds [31] which structure information can be effectively described by molecular fingerprints. Moreover, the calculation of molecular fingerprint only needs two-dimensional structure, which not only reduces the workload of molecular descriptor calculation and screening but also avoids the error transfer and accumulation in the process of molecular descriptor calculation. According to detecting the existence of specific structural segments in the molecular structure of drug compounds, these fragments then are encoded on the corresponding bits of the corresponding binary string through a substructure pattern of a predefined dictionary, and the molecular structure is transformed into an orderly digital fingerprint sequence. Specifically, the specific structural segment exists in the given drug molecules; the corresponding bit of the vector is set to 1, or 0 would be set. In this work, the chemical structure fingerprints set can be downloaded from the PubChem website (https://pubchem.ncbi.nlm.nih.gov/). There are 881 substructure information recorded in drug fingerprint. As a result, the molecular feature of the drug is 881 binary vectors.

### 2.3. Position-Specific Scoring Matrix

There exist many effective methods to transform protein sequences into multidimensional feature vectors such as using physicochemical of amino acids [32, 33] and using statistical distributions of amino acids [34, 35]. In this work, we adopt position-specific scoring matrix (PSSM) [36], which was adopted for exploring distantly related protein. PSSM is also widely adopted in previous work such as protein secondary structural prediction, protein binding site prediction, and protein subcellular localization. Through using a Position-Specific Iterated Basic Local Alignment Search Tool (PSI-BLAST) [37] to search and compare the homologous sequence of each target protein sequence, the homologous information of alignment sequence can be expressed as PSSM which construct is an *M* × 20 score matrix, where *M* rows are the total number of amino acid sequence and 20 columns represent the number of 20 amino acid. Here, a PSSM can be obtained according to the following formula:

(1) $$\begin{array}{c} {\text{PSSM}}_{P}=\left(M_{1},M_{2},\cdots ,M_{i},\cdots ,M_{20}\right), \end{array}$$

where *M*_*i*_ = (*M*_1,*i*_, *M*_2,*i*_, ⋯,*M*_*n*,*i*_, ⋯,*M*_*X*,*i*_)^*T*^, (*i* = 1, 2, ⋯, 20). *X* denotes the length of an amino acid sequence, and *M*_*n*,*i*_ is the mutation score which represents the probability of amino acid *i* residue to change into amino acid *j* in the process of biological evolution. In this experiment, the PSI-BLAST tool was employed to transform each protein sequence into a PSSM. For obtaining highly homologous sequence, the parameter of critical value of *e* value is set to 0.001 and maximum number of iterations is 3; other parameters were set to default values. For details on the use of PSI-BLAST can be obtained at https://BLAST.ncbi.nlm.nih.gov/BLAST.cgi.

### 2.4. GIST Feature Descriptor

The GIST feature which is a biological heuristic feature was first proposed by Oliva et al. [38]. The GIST feature can extract global feature information which plays an important role in scene image classification [39], and it has been proven to be feasible in objective recognition [40, 41] as well. In GIST algorithm, the processes mainly contain the following two steps to extract GIST feature:

*(1) Creating Gabor Filters*. In image processing, the Gabor filter can extract a feature from the gray-level images directly. In spatial domain, the two-dimensional Gabor filter is a Gaussian kernel function modulated by complex sinusoidal plane wave. The definition is as follows:

(2) $$\begin{array}{c} G\left(x,y\right)=\exp\left(-\frac{{x^{\prime }}^{2}+\gamma^{2}{y^{\prime }}^{2}}{2\sigma^{2}}\right)\exp\left(i\left(2\pi \frac{x^{\prime }}{\lambda }+\psi \right)\right), \end{array}$$

(3) $$\begin{array}{c} \text{Real part}:G\left(x,y\right)=\exp\left(-\frac{{x^{\prime }}^{2}+\gamma^{2}{y^{\prime }}^{2}}{2\sigma^{2}}\right)\cos\left(2\pi \frac{x^{\prime }}{\lambda }+\psi \right), \end{array}$$

(4) $$\begin{array}{c} \text{Imaginary part}:G\left(x,y\right)=\exp\left(-\frac{{x^{\prime }}^{2}+\gamma^{2}{y^{\prime }}^{2}}{2\sigma^{2}}\right)\sin\left(2\pi \frac{x^{\prime }}{\lambda }+\psi \right), \end{array}$$

where *x*′ = *x*cos*θ* + *y*sin*θ* and *y*′ = −*x*sin*θ* + *y*cos*θ*, *σ* is the standard deviation of Gauss function, *ψ* represents the phase shift, *λ* is the wavelength which value is specified in pixels, and *θ* is the direction of the parallel strips. Here, 32 Gabor filters were generated by adopting four scales and eight orientations.

*(2) Obtaining GIST Feature*. Given a training sample *T* which size is *a* × *b*. A sample *X* is divided into *n*_*a*_ × *n*_*b*_ blocks; each block is of equal size which construction can be defined as *a*^∗^ × *b*^∗^, where *a*^∗^ = *a*/*n*_*a*_and *b*^∗^ = *b*/*n*_*b*_. Each block is processed by employing a set of filter banks that contain 32 Gabor filters, and the processed features are combined to form the block feature, which is called a block GIST feature. The feature vectors of a block processed by each filter are averaged, and these feature vectors are combined into a row vector, which are connected in series to obtain the final GIST feature, we can be defined as follows:

(5) $$\begin{array}{c} G_{\text{vector}}=\left[\bar{G_{1}},\bar{G_{2}},\cdots ,\bar{G_{i}},\cdots ,\bar{G_{32}}\right], \end{array}$$

where $\bar{G_{i}}=\left(1/\left(a^{*}\times b^{*}\right)\right)\sum G_{i}\left(x,y\right)$ and the dimension of *G*_vector_ is 32 × *a*^∗^ × *b*^∗^. These feature vectors summarize the gradient information such as scale and orientation for different parts of a given sample [42, 43].

In this paper, each PSSM is divided into 16 regions 4 × 4 grids, using 32 Gabor filter banks with four scales and eight directions to extract the GIST feature of PSSM of each protein sequence. Finally, a 512 (16 × 32) dimensional GIST feature of each PSSM is obtained by connecting the 16 (4 × 4) average values of all 32 features maps.

### 2.5. Rotation Forest (RF) Classifier

The rotation forest algorithm was proposed by Rodriguez et al. [44]. This algorithm was based on the idea of feature transformation and focuses on improving the difference and accuracy of the base classifier. The sample set is randomly divided into *K* subsets before each subset is drawn, and the principal component analysis (PCA) method is used to perform feature transformation on the divided subsets which is aimed at maintaining the effective of data. It not only makes each subset different but also plays a certain role in data preprocessing. Hence, the rotation forest can further improve the diversity in the ensemble and enhance the accuracy of the base classifier.

Suppose that *w* = [*w*_1_, *w*_2_, ⋯, *w*_*n*_] contains *n* features of a sample. Let *W* be the training sample set which size is *N* × *n*, where *N* denotes the number of samples. Let *H* be the feature set, and the corresponding label be the *Y* = [*y*_1_, *y*_2_,⋯,*y*_*n*_]^*T*^. The feature set is randomly divided into *K* equal subsets. Suppose the number of decision trees is *L*, which can be denoted as *T*_1_, *T*_2_, ⋯*T*_*L*_, respectively. The construction steps of the rotation forest classifier are as follows:

1. Select the suitable parameter *K*, the feature set *H* is randomly divided into *K* subsets, each subset contains (*n*/*K*) features
2. Let *H*_*ij*_ denote the *j*th subfeature set of the training set, which is used to train the *i*th classifier *T*_*i*_. For each subset, a new training set *W*_*ij*_′ is generated after a bootstrap resampling with 75 percent of training set *W*
3. Apply principal component analysis (PCA) on *W*_*ij*_′ to produce the coefficients in matrix *P*_*ij*_, which is a matrix of *M* × 1. *P*_*ij*_ can be represented as *b*_*ij*_^(1)^, ⋯, *b*_*ij*_^(M_*j*_)^
4. The coefficients obtained in the matrix *P*_*ij*_ are constructed a sparse rotation matrix *S*_*i*_, which is shown as follows:

(6) $$\begin{array}{c} S_{i}=\left[\begin{array}{cccc} b_{i1}^{\left(1\right)},\cdots ,b_{i1}^{\left(M_{1}\right)} & 0 & \cdots & 0\\ 0 & b_{i2}^{\left(1\right)},\cdots ,b_{i2}^{\left(M_{2}\right)} & \cdots & 0\\ \vdots & \vdots & \ddots & \vdots \\ 0 & 0 & \cdots & b_{iK}^{\left(1\right)},\cdots ,b_{iK}^{\left(M_{K}\right)} \end{array}\right]. \end{array}$$

During the prediction period, given a sample *w*, let *d*_*ij*_(wS_*i*_^*b*^) be the probability which predicted whether *w* belongs to *y*_*i*_ by the classifier *T*_*i*_. Then, calculate the confidence of the class by means of the average combination, and the formula is as follows:

(7) $$\begin{array}{c} \lambda_{j}\left(w\right)=\frac{1}{L}\overset{L}{\underset{i=1}{\sum }}d_{ij}\left(\text{w}{\text{S}}_{i}^{b}\right). \end{array}$$

The test sample *w* will be assigned the category with the greatest possible.

## 3. Results and Discussion

### 3.1. Evaluation Criteria

Evaluation criteria play an effective role in evaluating the computational method. In this paper, we adopted the following criteria which include accuracy (Acc.), precision (Prec.), sensitivity (Sen.), and Matthews correlation coefficient (MCC). The definition is as follows:

(8) $$\begin{array}{c} \text{Acc}.=\frac{\text{TN}+\text{TP}}{\text{TN}+\text{TP}+\text{FN}+\text{FP}}, \end{array}$$

(9) $$\begin{array}{c} \text{Prec}.=\frac{\text{TP}}{\text{TP}+\text{FP}}, \end{array}$$

(10) $$\begin{array}{c} \text{Sen}.=\frac{\text{TP}}{\text{TP}+\text{FN}}, \end{array}$$

(11) $$\begin{array}{c} \text{MCC}=\frac{\text{TN}\times \text{TP}‐\text{FN}\times \text{FP}}{\sqrt{\left(\text{TN}+\text{FN}\right)\times \left(\text{TP}+\text{FP}\right)\times \left(\text{TN}+\text{FP}\right)\times \left(\text{FN}+\text{TP}\right)}}, \end{array}$$

where true negative (TN) represents the number of drug-target pairs that are classified as noninteracting pairs correctly, true positive (TP) denotes the count of drug-target pairs that are classified as interacting pairs correctly, false negative (FN) represents the number of samples that are classified as noninteracting pairs incorrectly, and false positive (FP) is the count of samples that are classified as interacting pairs incorrectly. Meanwhile, we computed the receiver operating characteristic (ROC) curve, the precision-recall (PR) curve, the area under a ROC curve (AUC), and the area under precision-recall curve (AUPR) for evaluating the performance of the proposed method visually. Due to the imbalanced dataset, it is more significantly to evaluate the proposed by employing AUPR in this study.

### 3.2. Performance of the Proposed Method

To better verify the performance of the proposed method in this study, we adopt the five-fold cross-validation method on different types of protein target datasets: *enzyme*, *ion channel*, *GPCRs*, and *nuclear receptor*. Specifically, the whole dataset would be separated into five parts that four datasets are used for training and one dataset is used for testing. By doing this, five training models would be generated for training datasets, and GIST feature vectors and the corresponding label would be the input data of the prediction model. Finally, the prediction score could be obtained for evaluating the interaction between drug and target protein. In addition, for the sake of fairness for all experiments in this work, the corresponding parameters *K* and *L* of the rotation forest-based classifier were set the same. The parameter *K* is set to be 10, and *L* is set to be 12. Here, *L* denotes the number of decision trees and *K* means the number of feature subsets. The prediction results of the proposed method by using the five-fold cross-validation method of *enzyme*, *ion channel*, *GPCRs*, and *nuclear receptor* datasets are listed in Tables 2-5.

**Figure 1:**
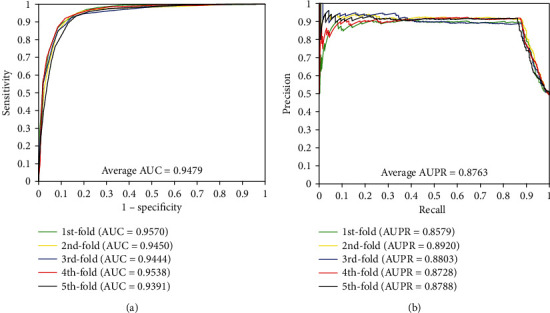
The curves obtained by the proposed method on the *enzyme* dataset: (a) ROC curves and (b) PR curves.

**Figure 2:**
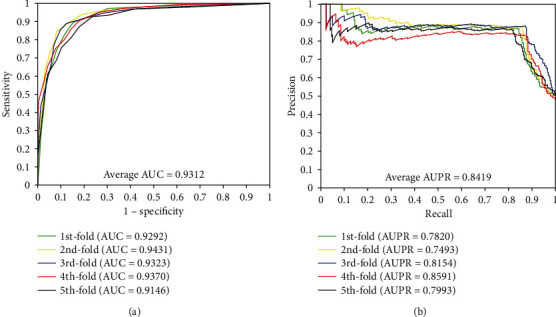
The curves obtained by the proposed method on the *ion channel* dataset: (a) ROC curves and (b) PR curves.

**Figure 3:**
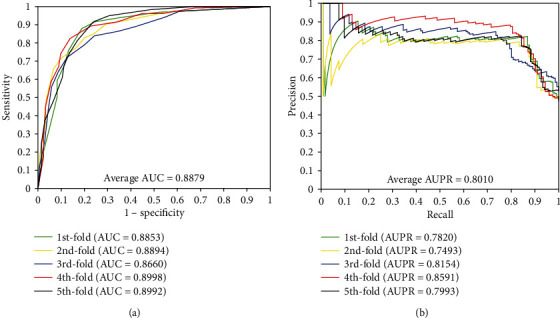
The curves obtained by the proposed method on the *GPCR* dataset: (a) ROC curves and (b) PR curves.

**Figure 4:**
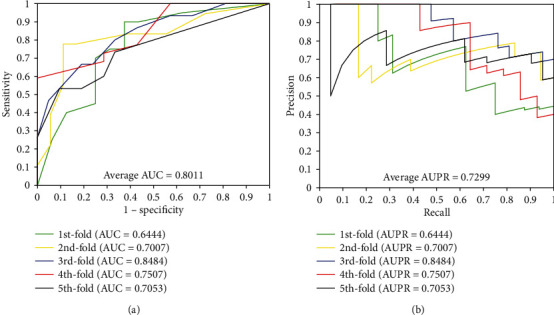
The curves obtained by the proposed method on the *nuclear receptor* dataset: (a) ROC curves and (b) PR curves.

When our method is used to predict DTI of the *enzyme* dataset, the results of average accuracy, precision, sensitivity, MCC, and AUC are 89.25%, 90.70%, 87.48%, 80.80%, and 0.9479, respectively. The standard deviations of these predicted results are 0.53%, 1.37%, 0.85%, 0.83%, and 0.0074, respectively. When employing our method to predict DTI of the *ion channel* dataset, the results of average accuracy, precision, sensitivity, MCC, and AUC come to be 85.93%, 86.35%, 85.38%, 75.84%, and 0.9312 and the standard deviations are 1.44%, 1.96%, 2.45%, 2.07%, and 0.0107, respectively. When exploring the *GPCRs* dataset, the prediction result of the average accuracy, precision, sensitivity, MCC, and AUC are 82.36%, 83.35%, 81.22%, 70.92%, and 0.8879, respectively. The standard deviations come to be 2.39%, 3.36%, 4.79%, 3.04%, and 0.0138, respectively. When predicting the interactions of the *nuclear receptor* dataset, we achieved the average result of accuracy, precision, sensitivity, MCC, and AUC of 73.89%, 73.82%, 75.83%, 60.15%, and 0.8011, respectively. It is noteworthy that the prediction result yields high standard deviations due to the samples of *nuclear receptor* dataset is only 90 which is smaller than the other three datasets, the standard deviations were 4.21%, 8.45%, 12.48%, 5.08%, and 0.0389, respectively. Furthermore, the values of AUPR were computed on *enzyme*, *ion channel*, *GPCRs*, and *nuclear receptor*, which achieved the result of 0.8763, 0.8419, 0.80101, and 0.7299, respectively. Meanwhile, the ROC curves and the PR curves of the proposed method of four datasets are shown in Figures 1-4 in order to better analyse the feasibility of the proposed method.

### 3.3. Comparison between RF Classifier and SVM Classifier

**Figure 5:**
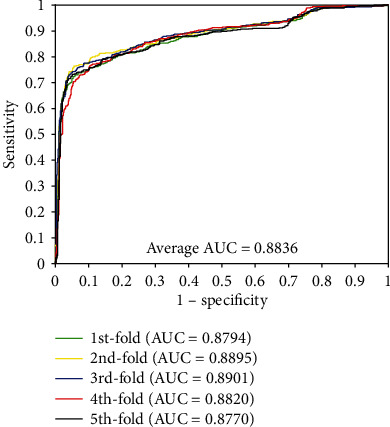
The ROC curves performed by SVM classifier on the *enzyme* dataset.

In order to further evaluate the prediction performance of the proposed method, we conducted the performance comparison between the RF classifier and the state-of-the-art support vector machine (SVM) classifier [45] by using the same feature descriptor vectors. We employed the five-fold cross-validation method for better analysis at the same time. The LIBSVM tool [46] was adopted to implement classification. We got the optimized parameters of the SVM classifier, and the parameter *c* is set to 15 and *g* is set to 30. The classification result of the *enzyme* dataset between the RF classifier and SVM classifier is listed in Table 6. It can be seen that the result of SVM classification of average accuracy, precision, sensitivity, MCC, and AUC of 81.83%, 83.34%, 79.54%, 70.23%, and 0.8836, and these standard deviation comes to be 0.64%, 0.78%, 1.16%, 0.83%, and 0.0059, respectively. From Table 6, we can see that the average results of the SVM classifier are lower than the performance of the proposed method. The prediction result also shows that the performance of the RF classifier is better than the performance of the SVM classifier when employing the same feature vectors as the input data. Furthermore, the parameter optimization of the RF classifier is more convenient than the SVM classifier. Meanwhile, the ROC curves of the SVM classifier are displayed in Figure 5.

### 3.4. Comparison with Other Methods

Until now, numerous computational methods have been proposed for predicting the DTI. In our study, we made a performance comparison between the proposed method and the other four existing methods that include NetCBP [47], Mousavian et al.'s [48], Li et al.'s [21], and RFDTI [49]; these methods were also employed the five-fold cross-validation on *enzyme*, *ion channel*, *GPCRs*, and *nuclear receptor* dataset, respectively. The differences of them were the different feature extractions and classifiers adopted. These comparison results are listed in Table 7. It can be seen from Table 7 that the results we obtained were improved than those previously proposed methods; the increases of average AUC values on *ion channel* and *GPCR*s datasets were 0.0141, and 0.0023, respectively. However, average AUC value of the *enzyme* and *nuclear receptor* datasets are little lower than previous works, which mainly caused by the scale of prediction samples and extraction method. Generally, the comparison results demonstrated that GIST feature extraction combined with the rotation forest classifier could improve the prediction performance of drug-target interactions effectively.

## 4. Conclusions

In this article, we reported a novel computational approach combines GIST feature, position-specific scoring matrix (PSSM), and rotation forest (RF) based classification to infer unknown DTIs on a large-scale. For further evaluating the prediction ability of the proposed method, we adopted the five-fold cross-validation method on golden standard datasets. When performing on *enzyme*, *ion channel*, *GPCRs*, *nuclear receptors*, the proposed method yielded the average accuracy of 89.20%, 85.93%, 82.36%, and 73.89%, respectively. In order to further assess the performance of the proposed model, we made a comparison between the proposed method and the state-of-the-art support vector machine classifier. We also compare with the previous models which were based on the same golden standard datasets. These extensive experimental results further illustrate that the proposed method is effective and robust in predicting drug-target interactions. We expect this proposed method to be a useful tool when predicting DTIs. In future work, we plan to use more advanced feature extraction method to improve the prediction ability of DTIs.

## Figures and Tables

**Table 1:** The statistic of four drug-target data.

Dataset	Drugs	Target proteins	Interactions
Enzyme	445	664	2926
Ion channels	210	204	1476
GPCRs	223	95	635
Nuclear receptors	54	26	90

**Table 2:** 5-fold cross-validation results were generated through the proposed method on the *enzyme* dataset.

Testing set	Accuracy (%)	Precision (%)	Sensitivity (%)	MCC (%)	AUC	AUPR
1	88.89	89.70	87.86	80.24	0.9570	0.8579
2	89.66	91.07	88.14	81.45	0.9450	0.8920
3	88.63	88.87	88.10	79.85	0.9444	0.8803
4	89.91	91.97	87.20	81.83	0.9538	0.8728
5	89.15	91.88	86.13	80.62	0.9391	0.8788
Average	**89.25 ± 0.53**	**90.70 ± 1.37**	**87.48 ± 0.85**	**80.80 ± 0.83**	**0.9479 ± 0.0074**	**0.8763 ± 0.0125**

**Table 3:** 5-fold cross-validation results were generated through the proposed method on the *ion channel* dataset.

Testing set	Accuracy (%)	Precision (%)	Sensitivity (%)	MCC (%)	AUC	AUPR
1	85.59	86.76	84.12	75.33	0.9292	0.8435
2	86.95	87.63	86.15	77.30	0.9431	0.8686
3	87.63	88.18	87.29	78.31	0.9323	0.8633
4	85.59	83.16	87.59	75.31	0.9370	0.8056
5	83.90	86.01	81.73	72.96	0.9146	0.8287
Average	**85.93 ± 1.44**	**86.35 ± 1.96**	**85.38 ± 2.45**	**75.84 ± 2.07**	**0.9312 ± 0.0107**	**0.8419 ± 0.0258**

**Table 4:** 5-fold cross-validation results were generated through the proposed method on the *GPCR* dataset.

Testing set	Accuracy (%)	Precision (%)	Sensitivity (%)	MCC (%)	AUC	AUPR
1	83.86	81.89	85.25	72.91	0.8853	0.7820
2	81.89	79.37	83.33	70.29	0.8894	0.7493
3	78.74	84.75	73.53	66.35	0.8660	0.8154
4	85.04	88.29	79.67	74.34	0.8998	0.8591
5	82.28	82.48	84.33	70.71	0.8992	0.7993
Average	**82.36 ± 2.39**	**83.35 ± 3.36**	**81.22 ± 4.79**	**70.92 ± 3.04**	**0.8879 ± 0.0138**	**0.8010 ± 0.0407**

**Table 5:** 5-fold cross-validation results were generated through the proposed method on the *nuclear receptor* dataset.

Testing set	Accuracy (%)	Precision (%)	Sensitivity (%)	MCC (%)	AUC	AUPR
1	75.00	76.92	62.50	60.78	0.7688	0.6444
2	80.56	78.95	83.33	68.62	0.8148	0.7007
3	69.44	81.25	61.90	56.69	0.8254	0.8484
4	72.22	60.00	85.71	58.61	0.8442	0.7507
5	72.22	72.00	85.71	56.06	0.7524	0.7053
Average	**73.89 ± 4.21**	**73.82 ± 8.45**	**75.83 ± 12.48**	**60.15 ± 5.08**	**0.8011 ± 0.0389**	**0.7299 ± 0.0762**

**Table 6:** 5-fold cross-validation results were generated by using the proposed RF classifier and SVM classifier on the *enzyme* dataset.

Testing set	Accuracy (%)	Precision (%)	Sensitivity (%)	MCC (%)	AUC
PSSM+GIST+RF					
1	88.89	89.70	87.86	80.24	0.9570
2	89.40	89.90	88.98	81.05	0.9450
3	88.63	88.87	88.10	79.85	0.9444
4	89.91	91.97	87.20	81.83	0.9538
5	89.15	91.88	86.13	80.62	0.9391
Average	**89.20 ± 0.49**	**90.46 ± 1.39**	**87.65 ± 1.07**	**80.72 ± 0.76**	**0.9479 ± 0.0073**
PSSM+GIST+SVM					
1	81.11	82.85	78.46	69.32	0.8794
2	82.65	83.65	81.53	71.31	0.8895
3	82.22	83.94	79.31	70.71	0.8901
4	81.28	82.23	79.24	69.53	0.8820
5	81.88	84.02	79.19	70.29	0.8770
Average	**81.83 ± 0.64**	**83.34 ± 0.78**	**79.54 ± 1.16**	**70.23 ± 0.83**	**0.8836 ± 0.0059**

**Table 7:** Comparison of the AUC values between the proposed method and other four existing methods on four datasets.

Dataset	Our method	NetCBP	Mousavian et al.	Li *et al*.	RFDTI
Enzyme	**0.9479**	0.8251	0.9480	0.9288	0.9172
Ion channels	**0.9312**	0.8034	0.8890	0.9171	0.8827
GPCRs	**0.8879**	0.8235	0.8720	0.8856	0.8557
Nuclear receptors	**0.8011**	0.8394	0.8690	0.9300	0.7531

## Data Availability

The data code can be obtained at https://github.com/TensorflowZhan/Program-Availability
